# How Lung Volume Recruitment Maneuvers Enhance Respiratory Function in Multiple Sclerosis Patients: A Quasi-Randomized Pilot Study

**DOI:** 10.3390/medicina59111896

**Published:** 2023-10-26

**Authors:** Claudia Enrichi, Martina Regazzetti, Błażej Cieślik, Cristiano Zanetti, Daniela D’Imperio, Elisa Compagno, Luisa Cacciante, Sara Federico, Giorgia Pregnolato, Mirko Zitti, Pawel Kiper

**Affiliations:** 1 Physical Medicine and Rehabilitation Unit, Azienda ULSS, 3 Serenissima, 30126 Venice, Italy; claudia.enrichi@aulss3.veneto.it (C.E.);; 2 Healthcare Innovation Technology Lab., IRCCS San Camillo Hospital, 30126 Venice, Italy; 3 IRCCS San Camillo Hospital, 30126 Venice, Italy; 4 CKR Centre de Kinésithérapie et Rééducation, 06000 Nice, France

**Keywords:** multiple sclerosis, respiratory complications, LVR maneuver, respiratory rehabilitation, lung capacity

## Abstract

*Background and Objectives*: In patients with multiple sclerosis (MS), a decrease in muscle strength can lead to limitations in pulmonary functions, potentially causing respiratory complications. To address these challenges, the lung volume recruitment (LVR) maneuver has emerged as a potential intervention. This study sought to evaluate the impact of a four-week LVR protocol on respiratory function in secondary progressive MS patients. *Materials and Methods*: In a quasi-randomized pre/post-controlled trial, 24 patients with secondary progressive MS were recruited. Participants aged 20–70 years with an EDSS score of 2 to 9 were alternately allocated to intervention (*n* = 12) or control groups (*n* = 12). The intervention group underwent a 4-week respiratory rehabilitation training focused on LVR, using a standardized cough machine treatment protocol twice daily. The control group received no respiratory intervention. Outcomes measured included forced vital capacity (FVC), maximal insufflation capacity (MIC), and peak cough flow (PCF), using turbine spirometry and other associated equipment. All measurements were taken at baseline (T_0_) and after 4 weeks (T_1_) by a blinded assessor. *Results*: For the intervention group, the mean difference pre/post-treatment in MIC (mL) was 0.45 (SD 1.13) (*p* = 0.02), and in MIC (%), it was 0.13 (SD 0.24) (*p* = 0.03). Compared to the control group (*n* = 10), the between-group mean difference for MIC (mL) was 0.54 (*p* = 0.02), and for MIC (%), it was 0.15 (*p* = 0.02). *Conclusions*: The short-term daily LVR protocol notably improved passive lung capacity, despite minimal changes in active lung capacity or cough force. The LVR maneuver offers promise for enhancing respiratory function, especially passive lung capacity, in secondary progressive MS patients. Further research should explore optimal treatment durations and frequencies for more extensive respiratory gains.

## 1. Introduction

Multiple sclerosis (MS) is an autoimmune, chronic, inflammatory, demyelinating, and multifocal disease affecting the central nervous system (CNS). It stands as the second leading cause of neurological disability in young adults and impacts approximately 2.5 million individuals globally. The manifestations of MS vary depending on the specific areas of the CNS affected [1]. Almost half of the patients diagnosed with MS experience mortality due to respiratory problems. These respiratory complications, characterized by alterations in ventilatory function, can emerge in the early stages of the disease and often remain asymptomatic. Consequently, without appropriate respiratory assessments, these issues often go unnoticed. They only become evident to clinicians when they manifest with symptoms at more advanced stages [2].

The pathology, interestingly, does not directly target the lungs or the cardiovascular system. Instead, its primary impact is on the respiratory muscles, causing them to become spastic and progressively weaker over time [3]. This weakening is not a sporadic or isolated event; it is a consistent and progressive deterioration in muscle strength. Even in ambulatory patients, this diminished strength is evident. Therefore, patients experience a reduction in lung volume, leading to challenges like ineffective coughing. This compromised ability to cough efficiently further results in impaired thoraco-pulmonary compliance and bronchial clearance. Such impairments can pave the way for the development of atelectasis, a condition where parts of the lungs collapse or do not inflate properly, further complicating the respiratory challenges faced by these patients.

This pathological condition evolves over the years, leading to restrictive syndrome and increasing the risk of developing respiratory failure [2,4]. In the clinical setting, there are two main types of respiratory rehabilitation interventions in MS: respiratory muscle training (RMT) and lung volume recruitment (LVR). Patients with MS suffer from alterations in respiratory function that can occur severely and at different stages of the disease [5]. Furthermore, respiratory failure and infectious diseases are the main causes of death in end-stage MS. One study showed that MS patients have an 11.7-times higher risk of dying from respiratory causes than the general population [6].

Respiratory disorders that can occur include respiratory muscle weakness and spasticity, sleep-disordered breathing, central sleep apnea, central respiratory dysregulation, or neurogenic swallowing disorders [5]. These deficits have also been described in the early stages of the disease in the absence of respiratory symptoms [7] and may contribute to worsening muscle weakness, fatigue, and cognitive disturbances [8]. Weakness of the respiratory muscles may lead not only to reduced lung volumes [4,5] but also to reduced cough efficiency, especially in the case of associated glottic dysfunction, exposing patients to reduced airway clearance and an increased risk of pneumonia [9]. Respiratory muscle weakness is measured by decreasing forced vital capacity (FVC), maximal inspiratory pressure (MIP), and maximal expiratory pressure (MEP). Respiratory failure in individuals with multiple sclerosis (MS) is linked to muscular factors rather than lung tissue (parenchyma) issues, with a distinct emphasis on more pronounced dysfunction in the auxiliary expiratory muscle.

This condition is determined by the fact that muscle paralysis in MS has an ascending course by first affecting the expiratory muscles, with more caudal innervation, and then the intercostals, and finally, the diaphragm (innervated by the C3–C5 phrenic nerve) [4]. In patients with mild-to-moderate disability and thus with more caudal impairment, the transmission speed of the impulses generated by the central conduction motor and directed to the diaphragm is altered [10]. The reduction in ventilatory volumes in MS patients is initially compensated by increasing the respiratory rate to maintain the correct levels of oxygen and carbon dioxide in the blood (pO_2_ and pCO_2_). As the disease progresses, however, a central adaptation mechanism to hypoventilation sets in, which induces hypercapnia (increased blood pCO_2_) and, in more advanced stages, hypoxemia (decreased blood pO_2_) [2]. This condition is further exacerbated by poor pulmonary distensibility, thus constituting restrictive syndrome [11]. In different studies, LVR was shown to have an immediate and statistically significant effect on assisted peak cough flow (PCF), with values increasing from 6% to 122% [12]. This increase in PCF cannot be defined as an improvement in the patient’s ability to cough independently but as an increase in the ability to cough during the LVR maneuver. One study [13] examined subgroups of patients defined by the baseline PCF value: In the groups of patients falling into the lowest three quartiles (baseline PCF < 190 L/min), the effect of LVR was statistically significant (*p* < 0.007). However, in the highest quartile (basal PCF > 190 L/min and mean basal PCF of 231.8 L/min), the difference between basal and assisted PCF was not statistically significant. This suggests that the effects of LVR may be greater in patients with a lower basal PCF, a theory that is also supported by other authors [14,15]. In addition to basal PCF, the presence of scoliosis has also been proposed as a potential variable that may influence LVR efficacy, as it reduces thoraco-pulmonary compliance [16]. There is a need in literature to develop further studies to understand the efficacy of LVR in respiratory function and to understand the underlying mechanisms. The present study aimed to assess the effectiveness of a four-week LVR maneuver in counteracting restrictive syndrome in subjects with secondary progressive MS. Specifically, based on the findings in scientific literature, the primary objective was to determine if the daily application of an LVR treatment protocol could influence respiratory function parameters.

## 2. Materials and Methods

### 2.1. Study Design and Participants

This study, designed as a quasi-randomized pre/post-controlled trial, targeted patients with secondary progressive MS. Following the participant enrollment guidelines established by Julious (2005) and Whitehead et al. (2015) [17,18], we consecutively recruited 24 patients admitted to IRCCS San Camillo Hospital in Venice, Italy for rehabilitation between January 2018 and January 2020. Notably, these individuals were not admitted to the facility due to specific respiratory problems; rather, their admission was for the purpose of neuromotor rehabilitation. Using an alternate allocation method, participants were sequentially divided: every second participant was placed in the intervention group, receiving respiratory rehabilitation training, while the others were placed in the control group, receiving no intervention [19]. Outcome measures were evaluated at the beginning (T_0_) and then after 4 weeks (T_1_) by an assessor who was blinded to the group allocations. This study enrolled patients who: (1) had secondary progressive multiple sclerosis, with the diagnosis being established with the McDonald criteria [20], (2) were between 20 and 70 years of age, and (3) had an Expanded Disability Status Scale (EDSS) score ranging from 2 to 9. Patients with secondary progressive MS were chosen for recruitment because of the condition’s relatively stable clinical trajectory, minimizing potential biases from acute rapid deterioration. Patients were excluded if they had experienced disease exacerbations within the three months prior to the protocol application; had concomitant respiratory diseases, such as asthma, COPD, pulmonary emphysema, or pulmonary fibrosis; used invasive or non-invasive ventilation or had cardiopathy; or exhibited dysphagia, indicated by a Functional Oral Intake Scale (FOIS) score below 5, meaning anything less than a complete oral diet of multiple consistencies.

The protocol, aligned with the principles of the Helsinki Declaration, was reviewed and approved by the Institutional Review Board of San Camillo Hospital (Venice, Italy) under reference number “protocollo 2018.01”. All participants provided written informed consent to partake in the research.

### 2.2. Intervention

The intervention group participated in a four-week program of respiratory rehabilitation training, whereas the control group did not receive any respiratory therapy or treatment inside the IRCCS San Camillo Hospital in Venice. Patients participated in two daily sessions for four weeks, five days a week, one in the morning and another in the afternoon, with a focus on LVR. The treatment approach included employing a cough machine (Model E-70 In-Ex Sufflator, Philips Respironics, Milan, Italy) to deliver air boluses up to the total lung capacity (TLC). The decision regarding whether to use an oronasal anesthesia mask or an oral interface during the incentive sessions was contingent upon the patient’s mouth’s orbicular muscles and buccinator functionality. Moreover, within these sessions, we integrated an antibacterial–antiviral humidifying filter (UmidVent) in conjunction with the cough machine. Each patient in the study adhered to standardized parameters. These parameters included a 5 s inspiratory time to ensure TLC was reached, a 5 s pause between insufflations to facilitate CO_2_ stabilization and prevent hyperventilation, maintaining a low flow rate for comfort (although the cough machine could accommodate higher rates for those with significant respiratory insufficiency), deactivating oscillations (as they were more relevant for bronchial unblocking in the presence of secretions), and adjusting inspiratory pressure between 40 cm H_2_O and 50 cm H_2_O, based on individual tolerance and lung condition, with a maximum limit of 50 cm H_2_O. Each session included several insufflation series, with the spacing between insufflations adjusted according to the individual patient’s tolerance while ensuring a consistent total of 35 insufflations per session. After the final insufflation in each series, patients held their breath for three seconds, followed by a one-minute break between series. Each session had a duration of about 15 min, with a 4 to 6 h interval between the morning and afternoon sessions.

### 2.3. Outcome Measurements

The study evaluated several outcomes related to respiratory health and functionality. FVC and its corresponding percentage (FVC%) measure the total volume of air that a person can forcefully exhale after a full inhalation. The FVC% provides a comparison of an individual’s FVC to expected values based on age, sex, and height [21]. Maximal insufflation capacity (MIC) and its associated percentage (MIC%) determine the maximum volume of air that the lungs can hold after a maximal inhalation [15]. The MIC% compares this to an expected standard, shedding light on thoracic–pulmonary compliance, which reflects how the lungs and chest wall expand and contract during breathing. PCF quantifies the maximum speed of air movement during a forceful cough, representing the efficiency of coughing in clearing the airways. To measure FVC, FVC%, and PCF, participants were equipped with an anesthetic face mask or an oral interface combined with a nose plug. This equipment was connected to a turbine spirometer (Model Pony FX Cosmed, Rome, Italy).

Each participant underwent three measurements, with the most optimal one selected for subsequent analysis. For the FVC measurement, subjects began with quiet breathing until respiratory volume stabilized. Subsequently, they performed a rapid, maximal inhalation, followed by a complete forced exhalation in accordance with established guidelines [22]. The FVC metric was assessed by executing a forceful cough after full inhalation [23]. To determine MIC, a cough machine was connected to an oronasal anesthesia mask, and the same spirometer used in previous measurements was employed. Participants underwent three to five consecutive insufflations tailored to their individual maximum inspiratory capacity. After reaching the tele-inspiratory phase, a swift, forceful exhalation was induced into the oronasal mask connected to the spirometer.

### 2.4. Data Analysis

Data were analyzed using JASP (Jeffreys’s Amazing Statistics Program) version 0.16.4 (University of Amsterdam, The Netherlands). Continuous variables were reported as the mean and standard deviation (SD), while categorical variables were expressed as counts and percentages. In the initial assessment of quantitative variables, we applied the Shapiro–Wilk normality test to each variable. Subsequently, we calculated the differences between the initial and final values of FVC, PCF, and MIC and assessed the normality of these differences. For variables with a normal distribution, we performed comparisons between the two groups using either the paired *t*-test for within-group comparisons or the unpaired *t*-test for between-group comparisons. To assess the extent of the differences, we computed Cohen’s *d* [24]. Values between 0.20 and 0.49 were categorized as small, those between 0.50 and 0.79 were deemed moderate, and any values of 0.80 or higher were labeled as large. Categorical variables were analyzed using the *χ*^2^ test. The threshold for statistical significance was set at *α* < 0.05.

## 3. Results

### 3.1. Participant Characteristics

**Figure 1:**
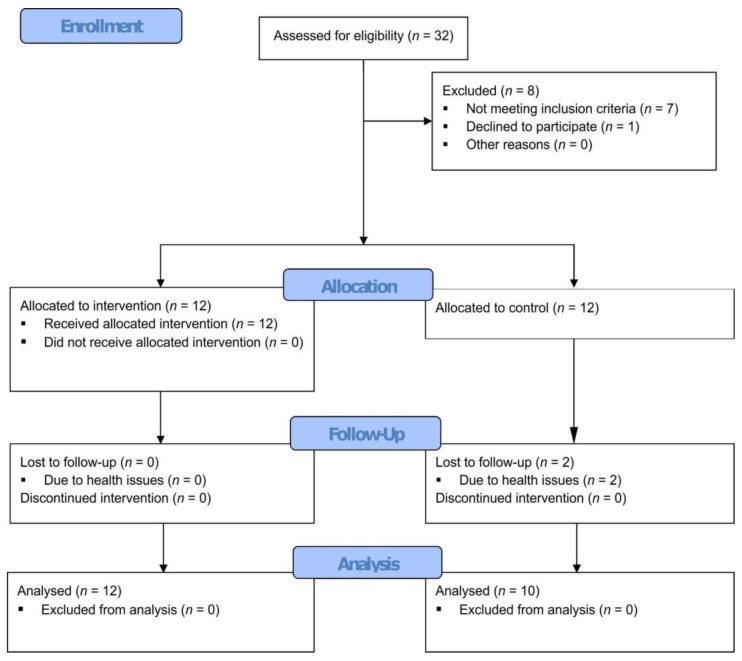
Study flow diagram.

Out of the 24 initial participants, 22 finished the study, with 12 in the study group and 10 in the control group. The control group had two drop-outs, both resulting from health concerns. The flow of the study can be visualized in Figure 1.

The two initial groups were comparable in terms of all variables, including age, sex, EDSS, BMI, initial FVC, PCF, and MIC. The baseline characteristics of the participants are presented in Table 1. In both groups, patients exhibited a high level of disability, with the study group having an average EDSS of 7.70 and the control group averaging at 7.40 (*p* = 0.38). No patient recorded an EDSS score below 6.00 (patient needed assistance to walk 100 m). Regarding BMI, most of the patients were of normal weight on average. When assessing basal forced vital capacity, 13 patients (54.17%) showed a deficiency, ranging from minor to moderately severe (50–59% and up to 80% of the predicted value, respectively). Additionally, five patients (20.83%) exhibited a severe or very severe decrease in FVC, with values registering below 49%, as detailed in Table 1.

### 3.2. Intervention Effects

The effects of the intervention, as well as the comparisons between groups, are presented in Table 2. In the assessment of respiratory outcomes between the intervention and control groups, distinct findings were observed in the MIC and MIC% metrics. For the intervention group, the MIC baseline value was 3.12 mL (SD = 1.19), which increased post-treatment to 3.57 mL (SD = 1.06), representing a 14.4% enhancement, with a Cohen’s d of −0.76 (*p* = 0.02). Similarly, MIC% showed an improvement from a baseline of 0.86 (SD = 0.28) to 0.99 (SD = 0.20) post-treatment, marking a 15.1% increase, with a Cohen’s d of −0.72 (*p* = 0.03).

In the control group, MIC started at 3.72 mL (SD = 1.55) and slightly decreased to 3.62 mL (SD = 1.45) post-treatment, while MIC% had a baseline of 91.10 (SD = 16.79) and decreased to 88.90 (SD = 16.01) post-treatment. The between-group comparisons underscored these variances, with MIC illustrating a significant mean difference of 0.54 (95% CI 0.10–0.98, *p* = 0.02) and MIC% denoting a mean difference of 0.15 (95% CI 0.03–0.28, *p* = 0.02), both favoring the intervention.

Regarding other parameters, such as FVC and PCF, while they were measured, they did not exhibit statistically significant changes post-intervention. For instance, in the intervention group, FVC values shifted from 2.47 mL (SD = 1.35) at baseline to 2.54 mL (SD = 1.33) post-treatment, while in the control group, FVC moved slightly from 3.16 mL (SD = 1.43) to 3.17 mL (SD = 1.33). Similarly, PCF in the intervention group changed from 267.42 mL/m (SD = 171.34) at baseline to 282.83 mL/m (SD = 157.23) post-treatment, and in the control group, it changed from 284.40 mL/m (SD = 140.75) to 305.60 mL/m (SD = 116.74).

## 4. Discussion

The present study evaluated the impact of a four-week LVR maneuver on restrictive syndrome in individuals with MS, specifically investigating whether daily LVR application could alter respiratory function parameters. In our study, implementing a daily LVR protocol over a short term did not result in a significant increase in FVC or PCF; however, there was a notable improvement in MIC in the study group, compared to the control group that did not undergo the protocol (*p* < 0.05). MS is an autoimmune and degenerative disorder affecting the central nervous system, and its primary impact on lung function stems from muscle weakness. Consequently, it seems plausible that improvements in forced vital capacity (FVC) and peak cough flow (PCF) may not be readily apparent, given their direct correlation with patient exertion.

MIC, on the other hand, is a parameter describing passive lung capacity and thoraco-pulmonary compliance, independent of muscle strength. We have seen how in MS patients, ventilatory insufficiency originates from the progressive decrease in respiratory muscle strength, which, over time, leads to a decrease in thoraco-pulmonary compliance, which, in turn, causes micro-atelectasis in the lung parenchyma. This mechanism consequently establishes a vicious circle whereby the decrease in compliance leads to an increase in respiratory work, which further strains the already deficient respiratory muscles [11,25,26,27]. Our hypothesis is that acting on the MIC, on compliance, through the LVR breaks this vicious circle, allowing the respiratory system to work in more optimal conditions, despite the progressive worsening of the respiratory musculature due to the disease.

The results of this study, in fact, provide information on the mechanisms through which the incentive of alveolar recruitment can help to improve and preserve the distensibility of the chest wall, acting on the range of motion of the cost-sternal and costovertebral joints, on the elasticity of the respiratory musculature, and on the elastic properties of the lung parenchyma. In addition, a small increase in MIC was found in patients performing LVR [16], and an increase in the difference between MIC and FVC over the duration of the study was also found. The MIC value also increased in the first 4 to 5 years after the start of the administration of the respiratory incentive maneuvers and then stabilized [28]. Our study showed that by insufflating air until TLC is reached through LVR, we act on two fronts: we preserve the lung parenchyma from micro-atelectasis due to hypoventilation and we keep the respiratory system elastic. These assumptions have been hypothesized to underlie the lower decline in respiratory function in patients treated with LVR [16,26,27,28,29]. Furthermore, this hypothesis was demonstrated through an MRI study that investigated the diffusion coefficient of He in the lungs and saw that a significant relative increase in the mean apparent diffusion coefficient (ADC) was observed in the waiting period between the two recruitment maneuvers [30].

To date, the optimal frequency of alveolar recruitment in the management of chronic respiratory failure remains an area of uncertainty and variability within medical literature. Some authors advocate for a once-daily approach [12,13,14,25], while others support a twice-daily regimen [28,31]. Conversely, some studies propose a three-times-daily protocol [16,25,31,32], and a subset of researchers does not explicitly specify a preferred frequency [26]. In the domain of clinical investigations, the selection of treatment frequency has resulted in a wide range of outcomes. Notably, a single daily treatment regimen has demonstrated enhanced PCF in patients afflicted with Duchenne syndrome [12]. It is also true that Duchenne muscular dystrophy follows a distinct pathology and course, typically manifesting initial symptoms in childhood. Early involvement of respiratory muscles is common, and there is no impairment of neural control within the central nervous system (CNS). Consequently, using this condition as a comparison to MS is not accurate. MS is a highly complex disease, necessitating the investigation of the optimal frequency of alveolar recruitment specifically in MS patients. In contrast, studies implementing a twice-daily regimen have exhibited a noteworthy increase in FVC, indicative of improved pulmonary function [31]. Furthermore, the three-times-a-day frequency has shown a notable rise in MIC [16]. These findings underscore the complexity of optimizing alveolar recruitment strategies, with outcomes varying based on the chosen frequency of intervention. As such, a comprehensive understanding of patient-specific needs and tailored approaches may be warranted to determine the most effective management strategy for chronic respiratory failure. Future research efforts should persist in examining this complex terrain in order to establish guidelines for clinical practice that are grounded in evidence. Clinical practice consistently emphasizes the importance of patients learning techniques to enhance cough strength—such as LVR and both manual and mechanically assisted coughing—during phases of clinical stability rather than when symptomatic. This proactive approach can be particularly effective. In this context, the notable increase in MIC among the intervention group highlights the potential clinical significance of LVR maneuvers for MS patients. Increased inspiratory capacity can mean an increase in lung volume and function, facilitating the removal of secretions. This improves quality of life by supporting daily activities and potentially reducing the breathing-related complications often associated with MS [32].

Our preliminary observations suggest that participants generally showed a favorable reception of the maneuvers. Over a prolonged period, in a specific cohort of individuals diagnosed with multiple sclerosis, the use of lung volume recruitment techniques correlated with a lower rate of deterioration in lung function and PCF [32]. Compared to alternative respiratory interventions available to patients with multiple sclerosis, alveolar recruitment maneuvers emerge as a prospective alternative characterized by efficacy, ease of implementation, and patient acceptability. However, considering the variability in severity and progression of multiple sclerosis, tailoring these maneuvers to meet the unique requirements of individual patients may be the most prudent course of action. Nonetheless, given the variability in MS severity and its progression, tailoring these maneuvers to individual patient needs might be the wisest approach.

### Limitations and Future Study Directions

In this study, there are several notable limitations that warrant discussion. Firstly, the small sample size represents a primary constraint, significantly impacting the ability to generalize the findings. Furthermore, a critical aspect of this article pertains to the relatively brief intervention duration, spanning just four weeks. This limited timeframe may not adequately capture the nuances of the condition, particularly given its chronic nature. Additionally, it is important to note that the assessment of outcomes occurs solely at the outset and after the four-week intervention period without any follow up assessment. Another significant limitation lies in the absence of a rehabilitation intervention within the control group. This omission poses challenges in assessing the clinical efficacy of LVR in comparison to the established gold standard. As we consider integrating these maneuvers into standard MS patient care, it is crucial to evaluate feasibility, pinpoint the optimal timing in the disease’s progression, and determine the appropriate frequency of application. It is also essential to note that while our study did not specifically delve into safety metrics, the safety and tolerability of any therapeutic intervention cannot be understated. Therefore, it is necessary to conduct additional research to explore the impacts of LVR on patients with MS.

## 5. Conclusions

In conclusion, in the study involving individuals with secondary progressive MS, a respiratory rehabilitation treatment conducted twice daily for four weeks, focusing on LVR and utilizing a cough machine, demonstrated notable improvements in MIC. However, it did not yield a significant effect on FVC. This suggests that the LVR maneuver holds promise as a therapeutic approach for improving respiratory outcomes, specifically targeting passive lung capacity in secondary progressive MS patients. In addition, considering the multifaceted nature of respiratory health in secondary progressive MS, future research could delve into potential correlations between LVR-based rehabilitation and other aspects of pulmonary function. Exploring patient-specific factors such as disease severity and baseline respiratory status may provide valuable insights into the tailored application of this intervention. Additionally, investigating the sustainability of the observed improvements beyond the initial four-week period could shed light on the long-term efficacy and durability of the LVR maneuver. This understanding is crucial for optimizing treatment protocols and maximizing the overall respiratory well-being of individuals with secondary progressive MS.

## Figures and Tables

**Table 1:** Participant baseline characteristics.

Variable	Total (*n* = 24)	Intervention Group (*n* = 12)	Control Group (*n* = 12)	*p*-Value
Age, mean (SD)	50.41(9.00)	49.42 (7.59)	51.60 (10.85)	0.59 ^a^
Male, *n* (%)	11 (45.83)	5 (41.67)	6 (50.00)	0.67 ^b^
EDSS, mean (SD)	7.58 (0.79)	7.70 (0.92)	7.40 (0.62)	0.38 ^a^
9, *n* (%)	2 (8.33)	2 (16.67)	0 (0.00)	-
8.5, *n* (%)	1 (4.16)	1 (8.33)	0 (0.00)	-
8, *n* (%)	8 (33.33)	4 (33.33)	4 (33.33)	1.00 ^b^
7.5, *n* (%)	2 (8.33)	0 (0.00)	2 (16.67)	-
7, *n* (%)	6 (25)	4 (33.33)	2 (16.67)	0.35 ^b^
6.5, *n* (%)	3 (12.50)	0 (0.00)	3 (25.00)	-
6, *n* (%)	2 (8.33)	1 (8.33)	1 (0.00)	1.00 ^b^
BMI, mean (SD)	22.81 (3.6)	22.38 (4.55)	23.34 (2.30)	0.55 ^a^
PCF, mean (SD)	275.13 (154.73)	267.42 (171.34)	284.40 (140.75)	0.81 ^a^
MIC, mean (SD)	3.39 (1.36)	3.12 (1.19)	3.72 (1.55)	0.42 ^a^
MIC%, mean (SD)	88.18 (22.93)	85.76 (27.56)	91.10 (16.79)	0.60 ^a^
FVC, mean (SD)	2.78 (1.39)	2.47 (1.35)	3.16 (1.43)	0.26 ^a^
FVC%, mean (SD)	71.23 (28.41)	66.67 (33.35)	76.70 (21.52)	0.42 ^a^
>80%, *n* (%)	9 (37.50)	4 (33.33)	5 (41.67)	0.67 ^b^
70–79%, *n* (%)	3 (12.50)	2 (16.67)	1 (8.33)	0.54 ^b^
60–69%, *n* (%)	5 (20.84)	2 (16.67)	3 (25.00)	0.62 ^b^
50–59%, *n* (%)	2 (8.33)	1 (8.33)	1 (8.33)	1.00 ^b^
35–49%, *n* (%)	2 (8.33)	1 (8.33)	1 (8.33)	1.00 ^b^
<35%, *n* (%)	3 (12.50)	2 (16.67)	1 (8.33)	0.54 ^b^
Smokers, *n* (%)	2 (8.33)	1 (8.33)	1 (8.33)	1.00 ^b^

EDSS: Expanded Disability Status Scale; BMI: body mass index; FVC: forced vital capacity; ^a^ according to the unpaired *t*-test; ^b^ according to the *χ*^2^ test.

**Table 2:** The effects of the intervention.

	Intervention Group (*n* = 12)			Control Group (*n* = 10)			Between-Group Comparison	
	Baseline	Post-Treatment	*p*-Value	Baseline	Post-Treatment	*p*-Value	Mean Difference (IC 95%)	*p*-Value
FVC, mL	2.47 (1.35)	2.54 (1.33)	0.13	3.16 (1.43)	3.17 (1.33)	0.81	0.06 (−0.11–0.23)	0.48
FVC, %	66.67 (33.35)	68.58 (31.58)	0.19	76.70 (21.52)	76.30 (17.17)	0.83	0.02 (−0.02–0.07)	0.32
PCF, mL/m	267.42 (171.34)	282.83 (157.23)	0.51	284.40 (140.75)	305.60 (116.74)	0.80	−5.78 (37.31–−83.61)	0.88
MIC, mL	3.12 (1.19)	3.57 (1.06)	0.02	3.72 (1.55)	3.62 (1.45)	0.64	0.54 (0.10–0.98)	0.02
MIC, %	0.86 (0.28)	0.99 (0.20)	0.03	91.10 (16.79)	88.90 (16.01)	0.34	0.15 (0.03–0.28)	0.02

FVC: forced vital capacity, PCF: peak cough flow, MIC: maximal insufflation capacity; values are expressed as the mean and SD.

## Data Availability

Data are available upon reasonable request to the corresponding author.
